# Heterogeneous Computing for Vertebra Detection and Segmentation in X-Ray Images

**DOI:** 10.1155/2011/640208

**Published:** 2011-08-09

**Authors:** Fabian Lecron, Sidi Ahmed Mahmoudi, Mohammed Benjelloun, Saïd Mahmoudi, Pierre Manneback

**Affiliations:** Computer Science Department, Faculty of Engineering, University of Mons, Place du Parc, 20 7000 Mons, Belgium

## Abstract

The context of this work is related to the vertebra segmentation. The method we propose is based on the active shape model (ASM). An original approach taking advantage of the edge polygonal approximation was developed to locate the vertebra positions in a X-ray image. Despite the fact that segmentation results show good efficiency, the time is a key variable that has always to be optimized in a medical context. Therefore, we present how vertebra extraction can efficiently be performed in exploiting the full computing power of parallel (GPU) and heterogeneous (multi-CPU/multi-GPU) architectures. We propose a parallel hybrid implementation of the most intensive steps enabling to boost performance. Experimentations have been conducted using a set of high-resolution X-ray medical images, showing a global speedup ranging from 3 to 22, by comparison with the CPU implementation. Data transfer times between CPU and GPU memories were included in the execution times of our proposed implementation.

## 1. Introduction

The general context of the present work is the cervical vertebra mobility analysis on X-ray images. The objective is to be able to automatically measure the vertebral angular movements. The issue is to determine the angles between adjacent vertebræ on radiographs characterized by 3 patient's positions: flexion, neutral, and extension.

In [1], Puglisi et al. describe a protocol for the analysis of cervical vertebra mobility applied to hospital diagnosis or to scientific research. They show that for the mobility analysis, the vertebra contour needs to be defined by a human operator. This operation can be performed on the original or the digitized radiograph. One contribution of this paper is to develop automated methods to provide such data.

One way to extract quantitative data is to segment the vertebræ by digital image processing. Nowadays, regular radiography of the spine is the cheapest and the fastest way for a physician to detect vertebral abnormalities. Furthermore, as far as the patient is concerned, this procedure is sure and noninvasive. Despite these advantages, the segmentation of X-ray images is very challenging due to their nature. They are characterized by very poor contrast, blended contours, and the human body complexity is responsible for the fact that some parts of the image could be partially or completely hidden by other organs. In a medical context, a key variable has to be taken into account: the time. It is crucial to develop efficient applications with a reduced execution time, especially in the case of urgent diagnosis. To do so, one can imagine to use a parallel-based architecture such as cluster, grid computing, graphics processing units (GPUs).

The GPUs represent an efficient solution to solve this problem. However, such a solution does not exploit the CPU multiple computing units (cores) present in the majority of computers. Moreover, the solution based on GPU is seriously hampered by the high costs of data transfer between CPU and GPU memories. To limit these constraints, we propose a parallel hybrid implementation which allows exploiting effectively the full computing power of heterogeneous architectures. Notice that heterogeneous architectures dispose of both multiple CPU and multiple GPU cores. The proposed implementation is applied on the most intensive step of vertebra segmentation method. Indeed, we develop a parallel hybrid implementation of the recursive contour extraction technique using Canny's criteria [2]. Our choice to parallelize this method is due to its noise robustness and its reduced number of operations. These factors allow applying the application on large sets of medical images and enable to have more precise results for vertebra extraction. Our work is especially dedicated to the use of large images databases. Therefore, our framework could be used in a medical context given the growing number of patients. Another application could be associated to the search and the navigation in large images and videos databases, such as in [3].

The remainder of the paper is organized as follows: related works are described in Section 2.  Section 3 presents the CPU implementation of the proposed method based on active shape model.  Section 4 discusses the use of GPU for image processing algorithms, while Section 5 is devoted to the parallel hybrid implementation of our approach, exploiting effectively the full computing power of heterogeneous architectures.  Section 6 presents the obtained results of vertebra extraction using a data set of medical images and compares the performance between CPU, GPU and hybrid implementations. Finally, Section 7 concludes and proposes further work.

## 2. Related Work

 One can find two kinds of related work for which vertebra segmentation and optimal edge detection in medical images are the fundamental processing steps: the first one is related to sequential solutions for vertebra extraction using CPUs, and the second is related to the use of GPU to accelerate image processing algorithms, which can be exploited for medical applications. 

### 2.1. Vertebra Segmentation on CPU

 If we study the segmentation approaches described in the literature, we can observe that their effectiveness depends on the related medical imagery modality. One can distinguish 3 types of modality: the conventional radiography (X-ray), the computed tomography (CT) and the magnetic resonance (MR).

With regard to the MR images, a watershed algorithm has been used in [4] to segment and reconstruct intervertebral disks. The idea is to provide preoperative data with an image-guided surgery system. The method uses a combination of statistical and spectral texture features to discriminate closed regions representing intervertebral disks. Recently, Huang et al. have used a learning-based approach applied to the vertebra detection and segmentation on MR images [5]. To this end, features such as Harr wavelet are extracted on images to train an AdaBoost learning algorithm. Finally, a normalized graph cut algorithm is used to segment the exact contour of the vertebræ. A similar approach has also been proposed for the vertebra segmentation in the context of CT images. In [6], lumbar vertebræ are segmented by the minimization of the graph cut associated to a CT image.

Still in this context, the active contour algorithm which deforms and moves a contour submitted to internal and external energies is applied in [7]. In this work, Klinder et al. provides a framework dedicated to the detection, identification, and segmentation of CT images for the computer-assisted surgery. Concerning the segmentation part, they use a constrained deformable model defined in [8]. In the same idea, the level set method, which makes an interface evolve in the image, has also been dedicated to the vertebra segmentation in [9, 10]. The main drawback of these methods remains the strong influence of an initialization close to the target.

To deal with X-ray images, methods only based on the image information are not adapted. The efficient methods for MR or CT images are not suitable for radiographs because of the blended contours. An exact segmentation needs additional details about the object of interest. For this reason, a template matching algorithm combined with a polar signature system has been proposed in [11]. Other model-based methods such as active shape model [12] and active appearance model [13] showed their effectiveness. Basically, an active shape model is a statistical model generated from a set of training samples. A mean shape model is computed and placed near the vertebræ of interest on the radiograph. ASM search applies deformations on this mean shape so that it corresponds to the real vertebra contour. An active appearance model is based on the same principle but introduces a model of the underlying distribution of intensity around the landmarks. In this paper, since we do not need the information about the texture, we decided to use active shape model to characterize and segment the vertebræ. ASM and AAM have been, respectively, used in [14–16] and [17–20] for the vertebra segmentation. However, the models used are global ones, that is, defined by several vertebræ. The interest of that model is to provide information about the curvature and the dependence between two vertebræ. Nevertheless, in the context of the vertebral mobility analysis, the global models cannot explain all the curvature variability since 3 particular patient's positions are studied. The only way to achieve the segmentation is to use a local vertebra model. However, in order to ensure an exact contour extraction, we need to precisely initialize the segmentation step by placing mean shape very close to the vertebræ of interest. In the literature, the generalized Hough transform (GHT) is often used for that matter. In [21], the authors try to take advantage of the GHT on radiographs in a fully automatic way, but they present a segmentation rate equal to 47% for lumbar vertebræ without providing information about the detection rate. Very recently, Dong and Zheng have proposed a method combining GHT and the minimal intervention of a user with only 2 clicks in the image [22].

### 2.2. GPU for Image Processing

Many image processing and rendering algorithms are known by their high consumption of both computing power and memory. Beyond of image rendering, most of image processing algorithms contain phases which consist of similar calculations between image pixels. These facts make these algorithms prime candidates for acceleration on GPU by exploiting processing units in parallel. In this category, Yang et al. implemented several classic image processing algorithms on GPU with CUDA [23]. OpenVIDIA project [24] has implemented different computer vision algorithms running on graphics hardware such as single or multiple graphics processing units, using OpenGL [25], Cg [26], and CUDA [27]. Luo and Duraiswami proposed a GPU implementation [28] of the Canny edge detector [29]. There are also some GPU works in medical imaging for new volumetric rendering algorithms [30, 31] and magnetic resonance (MR) image reconstruction on GPU [32]. Otherwise, there are different works for the exploitation of heterogeneous architectures of multicores and GPUs. Ayguadé et al. proposed a flexible programming model for multicores [33]. StarPU [34] provides a unified runtime system for heterogeneous multicore architectures (CPUs and GPUs), enabling to develop effective scheduling strategies.

Actually, our contribution is to propose firstly an original automated approach to detect the vertebra location in a radiograph which will be used for the initialization of the segmentation phase. Next, we develop a model-based segmentation procedure especially adapted to the vertebral mobility study. We also contribute with improving performance of vertebra segmentation in X-ray medical images, by implementing the most intensive step of the proposed approach on heterogeneous architectures composed of both CPUs and GPUs. Indeed, we propose a parallel hybrid implementation of edge detection step using Deriche-Canny method [2] that enables a better noise removing and requires a less number of operations than Canny method. This hybrid implementation allows an efficient exploitation of the full computing power of heterogeneous architectures (multi-CPU/multi-GPU) and enables to more improve performance than the GPU implementation described in [35].

## 3. General Framework

 In this paper, we propose an original approach for the vertebra segmentation in the context of the vertebral mobility analysis. Our method is actually based on the active shape model. Global statistical models generally used in the literature are not able to explain the spine curvature variability induced by the 3 positions: extension, neutral, and flexion. Therefore, we decided to use a local vertebra model implying an exact initialization of the ASM-based segmentation, that is, placing the mean shape close to the vertebræ of interest. For that matter, we need to locate the position of all the vertebral bodies. To this end, vertebra features are detected according to an original procedure. Actually, the anterior corners of each vertebra are located in the radiograph by an approach based on the edge polygonal approximation. Once we have extracted the vertebra position, we can start the segmentation procedure.

### 3.1. Learning

 The learning phase starts with the creation of a sample of images. In our case, we use X-ray radiographs focused on the cervical vertebræ C3 to C7 (see Figure 2). Actually, each item of the sample has to be described by an information, that is, the coordinates of some landmarks located on the item contour. These points need to correspond on the different shapes in the sample. The resulting marked vertebræ are not directly exploitable. Every shape in the sample has particular position and orientation. Building a relevant statistical model requires to align the shapes. To this end, we use an alignment approach based on the Procrustes analysis and detailed in [36].

### 3.2. Modelization

 As soon as all the vertebra shapes are aligned, they can be used to build the active shape model. To do so, the mean shape is first computed and then a group of other allowable shapes is derived by moving the landmarks in specific directions, obtained by a principal component analysis (PCA) of the data. We refer the reader to [12] for more detail about the modelization.

### 3.3. Initialization

 In order to initialize the segmentation procedure, the mean shape has to be placed close to a vertebra of interest. In [37], we proposed an original method to locate points of interest in a radiograph. Here, we use part of this work but we also detect the vertebræ by their anterior corners. First, a user is asked to mark out 2 points in the image to determine a region of interest (ROI) by the higher anterior corner of the C3 vertebra and the lower anterior corner of the C7 vertebra. Then, all the vertebral bodies are detected with a process composed of 4 steps: a contrast-limited adaptive histogram equalization to improve the image contrast, an edge detector, an anterior corner detection, and finally the vertebra localization. 

#### 3.3.1. Contrast-Limited Adaptive Histogram Equalization

 The X-ray images we deal with have very poor contrast. Before any further process, we need to improve the image quality. A simple histogram equalization has no impact on the radiographs. Therefore, we propose to use a specific method: the contrast-limited adaptive histogram equalization [38].

**Figure 1:**
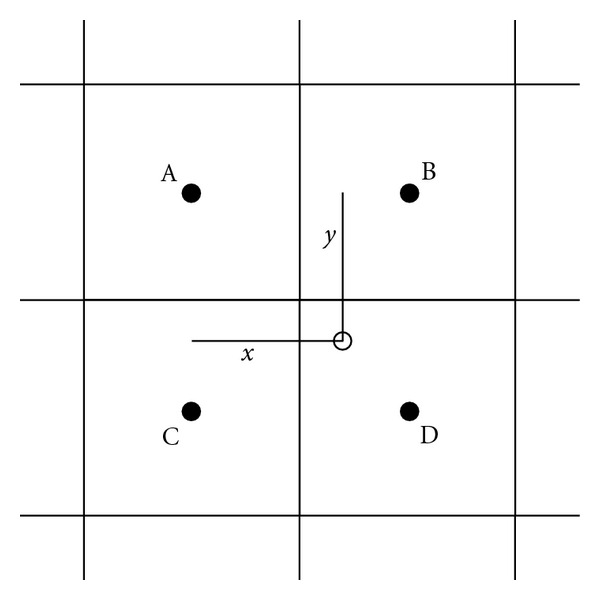
Image sample divided in 4 contextual regions.

The principle is to divide the image in contextual regions. In each of them, the histogram is equalized. Nevertheless, this transformation induces visual boundaries around the contextual regions. To get rid of this effect, a bilinear interpolation scheme is used. Let *A*, *B*, *C*, and *D* be the centers of contextual regions (see Figure 1). For a pixel *p*_*i*_ with an intensity *r*, we can write

(1) $$\begin{array}{cc} s & =\left(1-y\right)\left[\left(1-x\right)T_{A}\left(r\right)+xT_{B}\left(r\right)\right]\\ & \;+y\left[\left(1-x\right)T_{C}\left(r\right)+xT_{D}\left(r\right)\right] \end{array}$$

where *T*_*k*_ stands for the equalization transformation of the region *k*, and *s* for the new value of the pixel *p*_*i*_. 

Only applying this scheme is still dependent to the increase of the noise in the radiograph. One way to decrease it is to reduce the contrast improvement in the homogeneous areas. A contrast factor is then defined to limit the highest peaks in the equalized histogram. The pixels above this factor limit are uniformly redistributed in the histogram.

#### 3.3.2. Edge Detection

 The Canny edge detector, introduced in [29] allows to detect edges in an image by taking advantage of the information given by the intensity gradient. Let *I* be this image. The first step is to reduce the noise by removing isolated pixels. To this aim, the image is convolved with the Gaussian filter defined by (2).

(2) $$G\left(x,y\right)=\frac{1}{2\pi \sigma^{2}}e^{-(x^{2}+y^{2})/2\sigma^{2}}.$$

Next, the Sobel operator is applied on the resulting image. Let *A* be this image. The operator is based on a couple of masks defined by the relation (3). With this information, the gradient of a pixel can be computed by

(3) $$\begin{array}{c} G_{x}=\left(\begin{array}{ccc} -1 & 0 & 1\\ -2 & 0 & 2\\ -1 & 0 & 1 \end{array}\right)*A\;\;G_{y}=\left(\begin{array}{ccc} -1 & -2 & -1\\ 0 & 0 & 0\\ 1 & 2 & 1 \end{array}\right)*A, \end{array}$$

(4) $$\begin{array}{c} G=\sqrt{G_{x}^{2}+G_{y}^{2}}, \end{array}$$

Additional information about the gradient orientation is simply given by:

(5) $$\theta =\arctan\left(\frac{G_{y}}{G_{x}}\right).$$

Once the gradient has been computed for every pixel, only maxima have to be retained. High gradient intensity stands for high probability of edge presence. Finally, the last phase makes a hysteresis binarization. High and low thresholds are defined in such a way that, for each pixel, if the gradient intensity is

- lower than the low threshold, the point is rejected,
- greater than the high threshold, the point is part of the edge,
- between the low and the high thresholds, the point is accepted only if it is connected to an already accepted point.

#### 3.3.3. Corner Detection

 In this work, we propose to locate the vertebræ by firstly detecting some features: the anterior vertebra corners. To this end, we present a method based on the geometrical definition of a corner; that is, a point is considered as a corner if it is located at the intersection of two segment lines. The idea is to perform an edge polygonal approximation. Usually, works about the polygonal approximation detect the dominant points in the image and build a polygonal approximation. Here, we do the opposite by using the polygonal approximation to detect features in the image.

The Canny algorithm provides the edges on the image but only acts on the pixel values. In order to carry out the polygonal approximation algorithm, we need to define the contours as sets of points. Therefore, a simple contour tracking approach has been developed.

The algorithm used in this paper is the one proposed by Douglas and Peucker in [39]. This approach is based on the principle that a polyline represented by *n* points can be reduced in a representation with 2 points if the distance between the segment line joining the extremities of the polyline and the farthest point from this line is lower than a given threshold. The first stage concerns the selection of the extremities *E*_1_ and *E*_2_ of the polyline. Let *A* be the farthest point from the segment line ||*E*_1_*E*_2_|| and *d* the distance between the point *A* and ||*E*_1_*E*_2_||. Three scenarios are considered:

- if *d* ≤ *ϵ*, all the points situated between *E*_1_ and *E*_2_ are removed,
- if *d* > *ϵ*, the algorithm is recursively applied on 2 new polylines: ||*E*_1_*A*|| and ||*AE*_2_||,
- if there is no point between *E*_1_ and *E*_2_, the polyline is no longer reducible.

#### 3.3.4. Vertebra Localization

 Now that we have detailed how to detect corners in a general way, let us explain how to only detect the vertebra ones. Among all the corners detected by our approach based on the edge polygonal approximation, the ones describing a vertebra need to be distinguished.

**Figure 2:**
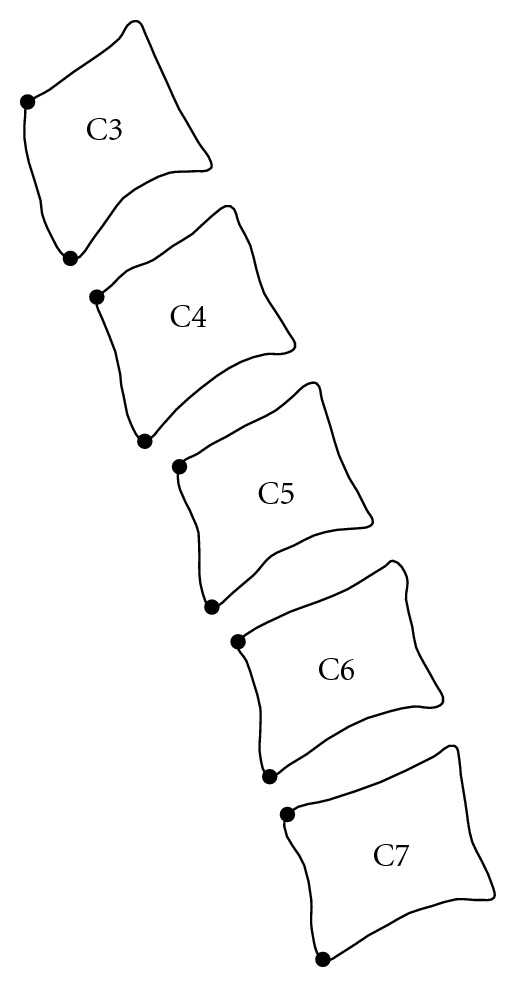
Landmarks for the spine curvature modelization.

The first stage of our procedure is to build a statistical model of the spine curvature in order to extract the mean shape. The landmarks of the model are the anterior vertebra corners. An illustration is given at the Figure 2. Notice that here, the goal is not to explain precisely the curvature but just to have a way to locate vertebra anterior corners. The next step brings a user to mark out the higher anterior corner of the C3 vertebra and the lower anterior corner of the C7 vertebra to define a ROI. Then, we perform an alignment between these two particular points and the mean shape of the spine curvature model. Finally, for each landmark, we search the closest corner detected by the approach based on the edge polygonal approximation. Note that a specific order has to be followed: from the top to the bottom of the image (the opposite could be considered). This order is crucial to avoid the algorithm swapping lower and higher corners of two successive vertebræ. 

### 3.4. Segmentation

The statistical model allowing to identify acceptable shapes of the object of interest is now defined. However, we still have to present how the search in the image is conducted during the segmentation. To this end, the grey level variation has to be locally evaluated around each landmark in the sample. Then, a mean profile of the texture (gradient intensity) can be deduced. After the initialization, a local analysis of the texture is carried out around each landmark of the initial shape. The goal is to find the best match with the mean profile previously determined. The distance used for the profile comparison is the Mahalanobis distance. This search implies that the landmarks are moved during the segmentation. The procedure is repeated until the convergence, that is, when the match between the current shape profile and the mean one is no more improved.

## 4. Image Processing on GPU

Image processing algorithms represent an excellent topic for acceleration on GPU, since the majority of these algorithms have sections which consist of a common computation over many pixels. This fact is due to the exploitation of the high number of GPU's computing units in parallel. As a result, we can say that graphics cards represent an efficient tool for boosting performance of image processing techniques. This section describes firstly the key factors of GPUs and the programming languages used to exploit their high power and secondly the proposed development scheme for image processing on GPU, based upon CUDA for parallel constructs and OpenGL for visualization.

### 4.1. GPU Programming

**Figure 3:**
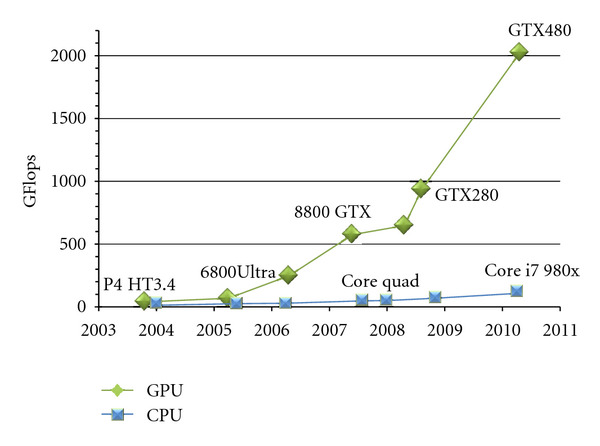
Computational Power: GPU versus CPU. Derived from [40].

Graphics processing units (GPUs) have dramatically evolved during last years as shown in Figure 3. This evolution makes them a very high attractive hardware platform for general purpose computation. For a better exploitation of this high power, the GPUs memory bandwidth has also significantly increased. Furthermore, the advent of GPGPU (general purpose graphics processing unit) languages enabled exploiting GPU for more types of application and not only for image rendering and video games. In this context, NVIDIA launched the API CUDA (compute unified device architecture) [27], a programming approach which exploits the unified design of the most current graphics processing units from NVIDIA. Under CUDA, GPUs consist of many processor cores which can address directly to GPU memories. This fact allows a more flexible programming model. As a result, CUDA has rapidly gained acceptance in domains where GPUs are used to execute different intensive parallel applications. 

### 4.2. Image Processing Model Based on CUDA and OpenGL

**Figure 4:**
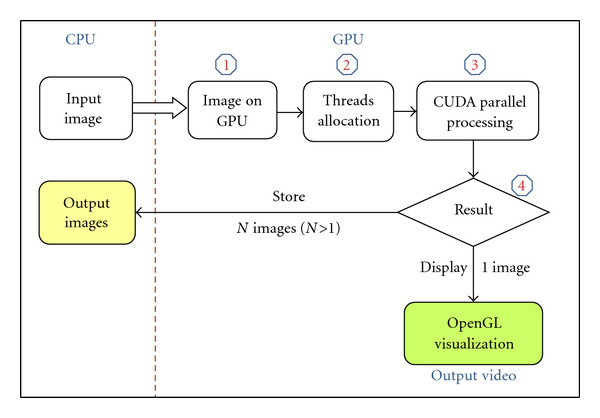
Image Processing on GPU based on CUDA and OpenGL.

We propose in this paragraph a model for image processing on graphics processors, enabling to load, treat, and display images on GPU. This model is represented by a scheme development based upon CUDA for parallel constructs and OpenGL for visualization, which reduces data transfer between device and host memories. This scheme is based on four steps (Figure 4):

- copy input data,
- threads allocation,
- parallel processing with CUDA,
- output results.

1. *Copy Input Data:* The transfer of input data (images) from host (CPU) to device (GPU) memory enables to apply GPU treatments on the copied image.
2. *Threads Allocation:* After loading the input data (images) on GPU memory, the threads number in the grid of GPU has to be selected so that each thread can perform its processing on one or a group of pixels. This allows threads to process in parallel on image pixels. We note that the selection of the number of threads depends on the number of pixels.
3. *Parallel Processing with CUDA:* The CUDA functions (kernels) are executed *N* times using the *N* selected threads in the previous step.
4. *Output Results: *After processing, results can be presented using two different scenarios.
  - *OpenGL Visualization:* The visualization of output images using the graphics library OpenGL is fast, since it exploits buffers already existing on GPU. Indeed, the compatibility of OpenGL with CUDA enables to avoid more data transfer between host and device memories. This scenario is useful when parallel processing is applied on one image only since we cannot display many images using one video output (one GPU disposes of one video output).
  - *Transfer of results:* the visualization with OpenGL is impossible in the case of applying treatments on a set of images using one video output only. In this case, the transfer of results (output images) from GPU to CPU memory is required. This transfer time represents an additional cost for the application.

## 5. Hybrid Implementation on Heterogeneous Architectures

 We presented in Section 3 the implementation details and steps of the proposed method of vertebra extraction on CPU. One disadvantage of this method is the computing time which increases significantly with the number of images and their resolution. Actually, the execution time of the edge detection is approximately 3 or 4 times greater than the time for histogram equalization and polygonal approximation. The ASM search procedure is not adapted for a parallel implementation due to the number of iterations which are dependent with each other. We proposed in [35] a solution based on the exploitation of the high power of GPUs in parallel. However, this solution does not exploit the CPU multiple computing units (cores) present in the majority of computers. Moreover, the solution based on GPU is hampered by the costs of data transfer between CPU and GPU memories. To reduce these constraints, we propose a parallel hybrid implementation which allows exploiting effectively the full computing power of heterogeneous architectures (multi-CPU/multi-GPU). This implementation is applied on the most intensive step of the vertebra segmentation method: edge detection. This section is presented in two parts: the first part describes our GPU implementation of edge detection step based on a recursive method. The second part describes the hybrid implementation of edge detection step on heterogeneous architectures.

### 5.1. GPU Implementation

This section describes the GPU implementation of edge detection step based on a recursive algorithm using Canny's design [2]. The noise truncature immunity and the reduced number of required operations make this method very efficient. This technique is based on four principale steps:

- recursive gradient computation (*G*_*x*_, *G*_*y*_).
- gradient magnitude and direction computation.
- non-maxima suppression.
- hysteresis and thresholding.

We note that the recursive gradient computation step applies a Gaussian smoothing before filtering the image recursively using two Sobel filters in order to compute the gradients *G*_*x*_ and *G*_*y*_. While the steps of gradient magnitude and direction computation, nonmaxima suppression, and hysteresis represent the same steps used for Canny filter described in Section 3.3.2.

The proposed GPU implementation of this recursive method is based on the parallelization of all the steps listed below on GPU using CUDA.

#### 5.1.1. Recursive Gaussian Smoothing on GPU

The GPU implementation of the recursive Gaussian smoothing step is developed using the CUDA SDK individual sample package [41]. This parallel implementation is applied on Deriche recursive method [2]. The advantage of this method is that the execution time is independent of the filter width. The use of this technique for smoothing allows to have a better noise truncature immunity which represents an important requirement for our application.

#### 5.1.2. Sobel Filtering on GPU

The recursive GPU implementation of this step is provided from the CUDA SDK individual sample package [41]. This parallel implementation exploits both shared and texture memories which allow to boost performance. This step applies a convolution of the source image by two Sobel filters of aperture size 3 in order to compute horizontal and vertical gradients *G*_*x*_ and *G*_*y*_ at each pixel. The GPU implementation is based firstly on a parallel horizontal convolution across the columns for computing *G*_*x*_ and secondly on a parallel vertical convolution across the lines for computing *G*_*y*_.

#### 5.1.3. Gradient Magnitude and Direction Computing on GPU

Once the horizontal and vertical gradients (*G*_*x*_ and *G*_*y*_) have been computed, it is possible to calculate the gradient magnitude (intensity) using (4) and the gradient direction using (5). The CUDA implementation of this step is applied in parallel on image pixels, using a GPU grid computing containing a number of threads equal to image pixels number. Thus, each thread calculates the gradient magnitude and direction of one pixel of the image.

#### 5.1.4. Nonmaxima Suppression on GPU

After computing the gradient magnitude and direction, we apply a CUDA function (kernel) which enumerates the local maxima (pixels with high gradient intensity) and deletes all nonridge pixels since local maxima are considered as a part of edges. We proposed to load the values of neighbors pixels (left, right, top, and bottom) in shared memory, since these values are required for the search of local maxima. The number of selected threads for parallelizing this step was also equal to image pixels number.

#### 5.1.5. Hysteresis on GPU

Hysteresis represents the final step to product edges. It is based on the use of two thresholds *T*_1_ and *T*_2_. Any pixel in the image that has a gradient magnitude greater than *T*_1_ is presumed to be an edge pixel and is marked as such immediately. Then, all the pixels connected to this edge pixel and that have a gradient intensity greater than *T*_2_ are also selected as edge pixels. The GPU implementation of this step is achieved using the method described in [28]. Notice that we exploit also the GPU's shared memory for a fast loading of connected pixels values.

### 5.2. Hybrid Implementation

The GPU implementation described below allowed to improve considerably the performance of edge detection step in the case of processing one image only, since results can be visualized quickly with OpenGL [35]. However, if we apply treatments on a set of medical images (as required in our proposed method of vertebra detection), the transfer of results (output images) from GPU to CPU memory will be required. This transfer time represents an important cost for the application. Thus, we propose to implement the edge detection step on a set of medical images, by exploiting the full computing power of heterogeneous architectures (multi-CPU/multi-GPU) that enables to have faster solutions, with less transfer of data between CPU and GPU memories, as the images processed on CPU do not require any transfer. The proposed implementation is based on the executive support StarPU [34] which provides a unified runtime system for heterogeneous multicore architectures. Therefore, our hybrid implementation of the edge detection step applied on a set of X-ray images can be described in three steps: loading of input images, hybrid processing with StarPU, and updating and storing results.

#### 5.2.1. Loading of Input Images

First, we have to load the input images in queues so that StarPU can apply treatments on images present on these queues.  Listing 1 summarizes this step.

**Loading of input images with StarPU.**
1 for ( *i* = 0; *i* < *n*; ++*i*) {*∖∖n*: number of images
2  img = cvLoadImage (Input_image};
3  starpu_data_handle img_handle;
4  starpu_vector_data_register(&img_handle);
5  queue = add(queue, img, img_handle);
6 }

Line 2 allows loading the image in main memory, lines 3 and 4 enable to allocate a buffer (handle) StarPU which disposes of the loaded image address. Line 5 is used to add this image and the buffer StarPU in a queue that will contain all the images to treat.

#### 5.2.2. Hybrid Processing with StarPU

Once the input images are loaded, StarPU can launch the CPU and GPU functions of edge detection (described, respectively, in Section 3.3.2 and 5.1) on heterogeneous processing units (CPUs and GPUs). StarPU is based on two main structures: the codelet and the task. The codelet defines the computing units that could be exploited (CPUs or/and GPUs), and the related implementations (Listing 2). The StarPU tasks apply this codelet on the set of images.

**The codelet StarPU.**
1  static starpu_codelet cl ={
2 .where = STARPU_CPU|STARPU_CUDA,  // CPU & GPU cores
3 .cpu_func = cpu_impl,   // define CPU fct
4 .cuda_func = cuda_impl,  // define GPU fct
5 .nbuffers = 1  // buffers number
6 };

In our case, each task is created and launched to treat one image in the queue. The scheduler of StarPU distributes automatically and effectively the tasks on the heterogeneous processing units. StarPU enables also an automatic transfer of data from CPU to GPU memory if tasks are executed on GPU. (Listing 3).

#### 5.2.3. Updating and Storing Results

When all the StarPU tasks are completed, the results of GPU treatments must be repatriated in the buffers. This update is provided by a specific function in StarPU. This function enables also to transfer data from GPU to CPU memory in the case of treatments applied on GPU.

**Submission of tasks to the set of images.**
1 while (queue != NULL)  {
2 task = starpu_task_create();  //Create task
3 task→cl = &cl;   //Define the codelet
4 task→handle = queue→img_handle;   //Define the buffer
5 task→handle.mode = STARPU_RW;   //Mode Read/Write
6 starpu_task_submit(task);   //Submit the task
7 queue = queue→next;   //Move to next image
8 }

## 6. Experimental Results

### 6.1. Segmentation

The validation of the cervical mobility evaluation is made by the validation of the segmentation approach. If we can be sure to know exactly the contour of the vertebræ, we can efficiently evaluate the angles between them. In order to do this, we use a sample of 51 radiographs coming from the NHANES II database of the National Library of Medicine (http://archive.nlm.nih.gov/proj/dxpnet/nhanes/nhanes.php). These images are the digitized versions of X-ray films collected during 1976–1980. Persons aged 25 through 74 were examined. Interesting data in this work are radiographs focused on the cervical vertebræ. Actually, we study the 5 vertebral bodies C3 to C7. Note that the resolution is the same for all images, that is, 1763 × 1755 pixels. We then chose randomly 51 X-ray films allowing the visual presence of the vertebræ C3 to C7. This way, we can fix the test set to validate the segmentation method.

**Figure 5:**
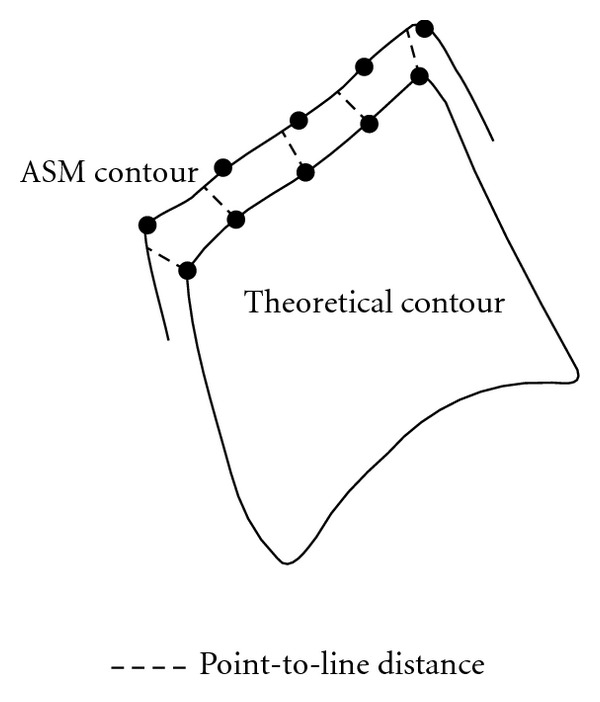
Point-to-line distance characterizing the error between a theoretical contour and an ASM-segmented contour.

One way to measure the segmentation error is to compute the distance between the ASM contour and a theoretical contour defined by a specialist. Therefore, a *gold standard* has been defined for the 51 radiographs of the test set. The chosen distance for measuring the segmentation error is the point-to-line distance. Used in [15, 16], the principle is to compute the length of the perpendicular dropped from each landmark of the theoretical contour to the spline evaluated between the landmarks of the ASM contour. A visual representation of the point-to-line distance is provided at the Figure 5.

 Further in this paragraph, we present statistical results on the segmentation error. The reader will find the mean error (in px) for the sample of 51 radiographs, the median (in px), and finally the failure rate. These indicators are computed at each vertebra level (from C3 to C7). Let us remark that the segmentation error is given in pixels. However, the scanner used by the NLM to digitize the radiographs was of 146 dpi. Therefore, we can consider that 1 px is approximately equal to 0.2 mm. In order to determine the failure rate, we followed the example presented in [16]. The segmentation error is divided in success and failure distribution. Therefore, we consider as a failure any error greater than 3 standard deviations from the mean of the success distribution.

Before the analysis of the segmentation results, we need to measure the quality of the initialization based on the detection of the vertebræ in the radiograph. As we previously noticed, the goal of detecting corners in cervical spine radiographs is to initialize the mean shape of the ASM search. In [37], we showed the benefits of the polygonal approximation dedicated to the points of interest detection by comparing it with the Harris detector [42]. The Harris and Stephen's definition of a corner uses the information of the Hessian matrix of grey level intensities. This detector is based on the local autocorrelation matrix of a signal on a region defined around each point, which measures the local changes of the signal in different directions. However, the results show that in the particular context of cervical spine radiographs, the intensity gradient information is not useful for detecting the points of interest. Actually, we demonstrate the interest of using a geometrical definition of a corner for its detection. Another advantage of the polygonal approximation is that once the Canny parameters have been chosen, only one parameter remains to be fixed: the threshold *ϵ*. Furthermore, there is no influence between the Canny parameters and the one of the polygonal approximation.

In this section, we evaluate the influence of the initialization on the results.  Table 1 shows the segmentation results with an initialization totally accomplished by a user. In fact, it was asked him to mark out manually all the vertebræ on the radiograph. We used a distinctive model for each vertebra level. In the literature [17–20], the models used are global ones. Their advantage is to bring information about the spine curvature, but they cannot efficiently accomplish a local segmentation. The only way to do this is to use a local vertebra model, but it requires a precise initialization close to the object of interest. The results of the Table 1 show the advantage to use such a model. The segmentation error is about 2.90 px and the percentage of failures is more than acceptable for each vertebra level (compared to the literature, see, for instance [15, 17–20]).

We could have stopped the experiments here, but it is not conceivable to ask a user to mark out all the vertebræ on every image he has to segment. For this reason, one of the contributions of this paper is to propose a semi-automatization of the ASM initialization. The data related to the Table 2 present the results based on this automated initialization. The analysis of the table demonstrates two particular trends. First, if we consider the mean segmentation error, we notice that its value is slightly increased in comparison with the Table 1. A meticulous analysis permits to target the step of the procedure responsible for this phenomenon. In fact, the results degradation is due to the points of interest detection by the polygonal approximation. Nevertheless, this effect is minimal if we look at the results of the Table 2. 

**Figure 6:**
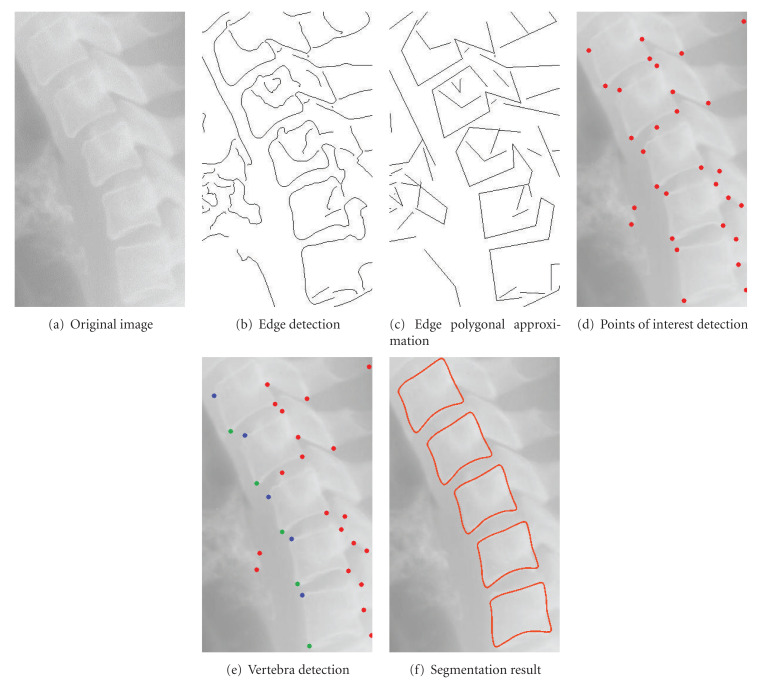
Illustration of the whole framework for the segmentation.

A particular limitation of our approach could arise in a specific case. If two vertebræ are merged, the corner detection could confuse a higher corner of a vertebra with the lower corner and the adjacent vertebra. Finally, we give the user a visual illustration of the whole framework to perform the vertebra segmentation at the Figure 6.

### 6.2. Performance

On the one hand, we can say that the quality of the vertebra segmentation remains identical since the procedure has not changed. Only the architecture and the implementation did. On the other hand, the exploitation of heterogeneous architectures (multi-CPU/multi-GPU) in parallel for vertebra extraction enabled to accelerate the computation time. This acceleration is due to the hybrid implementation for edge detection step based on a recursive method using Deriche-Canny method. This fact allowed to apply our proposed method on large sets of X-ray medical images in order to have more precision for vertebra detection results.

**Figure 7:**
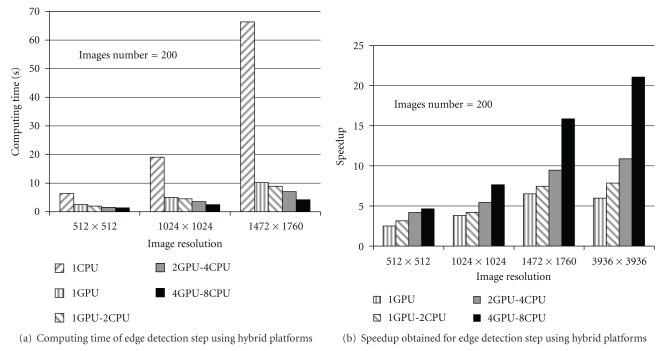
Performance of recurive edge detection using heterogeneous architectures.

Figure 7(a) presents the comparison of the computing times between sequential (CPU), parallel (GPU), and hybrid (multi-CPU/multi-GPU) implementations of the edge detection step, applied on a set of 200 images using different resolutions.  Figure 7(b) shows the speedups obtained with these implementations. The accelerations presented at Figure 7 are due to two level of parallelism: a low-level and a high-level parallelization.

- A low-level parallelization by porting the edge detection step on GPU (parallel processing between pixels in image: intraimage parallel processing).
- A high-level parallelization (interimages parallel processing) enabling to exploit simultaneously both CPUs and GPUs cores so that each core treats a subset of images.

Experimentations have been conducted on several platforms, that is, GPU Tesla C1060 and CPU Dual core:

- CPU: Dual Core 6600, 2.40 GHz, 2 GB RAM of Memory.
- GPU: NVIDIA Tesla C1060, 240 CUDA cores, 4 GB of Memory.

## 7. Conclusion

In this paper, we proposed a framework for vertebra segmentation based on a local statistical model. An original process in order to locate vertebræ in a radiograph has been developed. The principle is to detect features characterizing the vertebræ: the anterior corners. The extraction procedure is composed of 4 steps: a contrast-limited adaptive histogram equalization to improve the image contrast, an edge detection, an anterior corner detection, and finally the vertebra localization.

Generally, the computation time and noise immunity truncature represent the most important requirements in medical image processing and specifically for our application. The graphics processors provided a solution by exploiting the GPU's computing units in parallel. However, this solution is hampered by the costs of data transfers between CPU and GPU memories. Thus, we proposed a parallel hybrid implementation of the recursive edge method using Deriche-Canny approach. This implementation allowed to exploit the full computing power of heterogeneous architectures. Moreover, this solution requires a less transfer of data between CPU and GPU memories, as the treatments on CPU do not require any transfer.

As future work, we plan to develop a fully automatic segmentation approach based on a learning method such as support vector machine (SVM). The main issue is to find an efficient descriptor to train the supervised model. We also aim to provide an automatic parallel implementation exploiting the full computing power of hybrid architectures. This implementation could choose automatically the processing units for each step of our medical application. Thus, the most intensive steps (initialization: edge detection) would be implemented on heterogeneous platforms (multi-CPU/multi-GPU), and the less intensive or not parallelizable steps (learning, modelization, and segmentation) would exploit the CPU multiple cores (multi-CPU).

## Figures and Tables

**Table 1:** Statistical results on the error segmentation: local vertebra model (manual initialization).

Vert.	Mean (px)	Median (px)	Fail. (%)
C3	2.95	2.30	7.84
C4	2.63	2.43	1.96
C5	2.74	2.20	3.92
C6	2.98	2.65	3.92
C7	3.11	2.54	1.96

**Table 2:** Statistical results on the error segmentation: local vertebra model (automated initialization).

Vert.	Mean (px)	Median (px)	Fail. (%)
C3	2.97	2.36	7.84
C4	3.74	2.42	7.84
C5	2.86	2.34	5.88
C6	3.48	2.73	9.80
C7	3.27	2.50	5.88
